# Renal Function after Reduction in Cadmium Exposure: An 8-Year Follow-up of Residents in Cadmium-Polluted Areas

**DOI:** 10.1289/ehp.1103699

**Published:** 2011-10-25

**Authors:** Yihuai Liang, Lijian Lei, Johan Nilsson, Huiqi Li, Monica Nordberg, Alfred Bernard, Gunnar F. Nordberg, Ingvar A. Bergdahl, Taiyi Jin

**Affiliations:** 1 Department of Occupational Health and Toxicology (Key Laboratory of Public Health Safety, Ministry of Education of China), School of Public Health, Fudan University, Shanghai, People’s Republic of China; 2 Occupational and Environmental Medicine, Department of Public Health and Clinical Medicine, Umeå University, Umeå, Sweden; 3 Institute of Environmental Medicine, Karolinska Institutet, Stockholm, Sweden; 4 Unit of Toxicology, Université catholique de Louvain, Brussels, Belgium

**Keywords:** albumin, β_2_-microglobulin, cadmium, environmental exposure, *N*-acetyl-β-d-glucosaminidase, renal function

## Abstract

Background and objective: Long-term exposure to cadmium (Cd) causes renal dysfunction, but the change in renal function with exposure is unknown. We assessed the evolution of Cd-induced renal effects after a reduction in dietary exposure to Cd in rice.

Methods: Four hundred twelve residents in previously Cd-polluted and nonpolluted areas were examined twice, in 1998 and in 2006. Changes in blood Cd, urinary Cd, and kidney function [*N*-acetyl-β-d-glucosaminidase (NAG), β_2_-microglobulin, and albumin in urine] were measured.

Results: In the most polluted area, mean blood Cd was 8.9 μg/L and 3.3 μg/L in 1998 and in 2006, respectively, and urinary Cd was 11.6 and 9.0 μg/g creatinine. Urinary albumin in 1998 increased with urinary Cd, but no such exposure–response relation appeared for 2006 albumin versus urinary Cd 1998, indicating recovery. Other biomarkers of kidney function were also elevated in 1998. Partial recovery was observed for NAG among women and was suggested for β_2_-microglobulin among young individuals. The probability of having β_2_-microglobulin levels above the 95th percentile in 2006 was high in those with elevated β_2_-microglobulin in 1998 [odds ratio (OR) = 24.8; 95% confidence interval (CI): 11.2, 55.3] compared with albumin (OR = 3.0; 95% CI: 1.2, 7.5) and NAG (OR = 2.6; 95% CI: 1.6, 4.4).

Conclusions: Results suggest that a Cd-mediated increase in urinary albumin excretion is reversible upon substantial reduction of exposure. For markers of tubular effects, we observed a tendency toward improvement but not complete recovery. Data from repeated observations suggest that β_2_-microglobulin may be more informative than NAG as an indicator for an individual’s future tubular function.

Cadmium (Cd) has a very long biological half-life in humans (10–30 years). Occupational or environmental exposure to Cd can cause different health effects, one of the most prominent being kidney damage as a result of Cd accumulation in the kidney (Nordberg et al. 1971, 2007).

The evolution of Cd-related renal effects after changes in exposure conditions (i.e., cessation of or reduction in Cd exposure) is now being discussed in the scientific literature. An issue of major concern is whether renal effects of Cd are permanent, or whether renal function may recover after exposure ceases. Some authors have observed that severe renal tubular damage is irreversible in workers [urinary Cd (UCd) > 20 μg/g creatinine] previously exposed to high levels of Cd in the workplace (Jarup et al. 1993; Roels et al. 1997). Iwata et al. (1993) reported that renal dysfunction in individuals environmentally exposed to Cd via food became worse over time, even though exposure decreased from > 200 μg/day to half that value 14 years later, with a corresponding reduction in UCd from 8.5 to 6 μg/g creatinine. Hotz et al. (1999), on the other hand, reported that mild and early forms of renal dysfunction improved in a population that had been environmentally exposed to Cd: mean UCd excretion and blood Cd (BCd) at baseline were 8.9 nmol/24 hr and 9.4 nmol/L (~ 1 μg/L), respectively, for men, and 8.8 nmol/24 hr and 10.9 nmol/L for women. We previously published data from a 3-year follow-up (1995–1998) of 148 individuals who stopped consuming rice with high Cd content, including biomarkers of kidney function at baseline and after exposure ceased (Wu et al. 2008). The geometric mean (GM) of UCd among these individuals was 3.7 μg/g creatinine at baseline and 5.5 μg/g creatinine 3 years later (*p* = 0.064). The results indicated that the Cd-induced renal dysfunction might be reversible if baseline exposure is relatively low (UCd < 10 μg/g creatinine) before exposure decrease but may be irreversible or increased among those with higher exposure at baseline.

Here, we present data from 1998 and 2006 on 412 individuals from the same population as in the previous 3-year follow-up (Wu et al. 2008). Our aim was to evaluate changes in the prevalence of impaired kidney function after cessation of exposure to contaminated rice in relation to the initial exposure level and other characteristics such as age and sex. We measured three different biomarkers of kidney function [urinary *N*-acetyl-β-d-glucosaminidase (NAG), β_2_-microglobulin, and albumin] and also evaluated the ability of each biomarker to predict future impairment in the same individual.

The present study was part of a larger prospective study that also evaluated bone effects after reduction in Cd exposure (Chen et al. 2009a, 2009b). Results of that study suggested that effects of Cd on bone persisted over an extended period, resulting in a decline in bone mineral density and an increased prevalence of osteoporosis.

## Materials and Methods

*Study area and subjects.* The study area and subjects at baseline have been described previously (Jin et al. 1999, 2002, 2004a, 2004b). In brief, three areas in the Zhejiang Province, China, were studied: Jiaoweibao (highly polluted), Nanbaixiang (moderately polluted), and Yantou, where no particular pollution had occurred (nonpolluted). In the polluted areas, water contaminated by Cd-containing waste from a local Cd refinery had been used to irrigate farming areas for about 30 years, resulting in the contamination of locally grown rice. This rice was the major source of Cd intake until 1995, when residents stopped consuming locally grown rice. In 1996 residents of Jiaoweibao stopped producing rice and began eating commercially available rice from other (nonpolluted) areas. Although residents continued to consume locally grown vegetables, Cd intake was minimal compared with their previous intake from rice. In Nanbaixiang, the moderately polluted area, consumption of locally produced rice ended in the late 1990s because of rapid industrial and economic development in the Wenzhou region. Residents of Yantou, an area with similar living habits and socioeconomic conditions located about 40 km away from the contaminated areas, were also recruited into the study. Renal function of individuals from all three areas was assessed in 1995 (Nordberg et al. 1997) and 1998 (Jin et al. 2002).

In late 2006 we followed up participants in the studies mentioned above. The Cd content of rice produced in the highly polluted area was as high as 3.7 mg Cd/kg in 1995 (Nordberg et al. 1997), and thus we considered the consumption of rice grown locally as the most important determinant of Cd exposure. The ChinaCad study (Jin et al. 1999, 2002, 2004a, 2004b) showed that in 1998 most of the examined residents in the highly polluted area had BCd > 5 μg/L and UCd > 5 μg/g creatinine, despite being advised by local health authorities to consume rice from nonpolluted areas beginning in 1996. Cd exposure was associated with renal damage manifested by excessive excretion of urinary β_2_-microglobulin, albumin, NAG, and retinol-binding protein.

A total of 790 residents (488 women, 302 men) ≥ 35 years of age were surveyed in 1998, and 497 of them were followed up in 2006 (Chen et al. 2009a, 2009b). Among those who did not participate in the 2006 survey, 60 were dead, 9 were unavailable because of work outside the study areas, 57 had moved out of the study areas, and 167 declined to participate. After excluding individuals who were > 80 years old, had systolic blood pressure > 170 mmHg, or were on any physician-prescribed medication, a total of 412 subjects were available for the present study.

*Sample collection and bioanalysis.* Consistent with the same strict sampling protocol used in the 1998 study (Jin et al. 1999, 2002, 2004a, 2004b), blood and urine samples were collected and frozen at –20°C in the local laboratory on the same day. After all samples were collected, they were transported to Fudan University and stored at –80°C until analysis.

BCd and UCd were measured by graphite-furnace atomic absorption spectrometry using standard addition, as described by Jin et al. (2002). β_2_-Microglobulin and albumin were measured by enzyme-linked immunosorbent assay (ELISA). Urinary NAG was analyzed as previously described by Tucker et al. (1975). Creatinine in serum (available only in 2006) and urine were determined by the Jaffe reaction method (Hare 1950). To adjust the urine spot samples for dilution, all urine parameters were standardized to the concentration of creatinine in urine [for details of analytical performance, see Supplemental Material (http://dx.doi.org/10.1289/ehp.1103699)]. Glomerular filtration rate (GFR; in milliliters per minute per 1.73 m^2^) was estimated using the Chronic Kidney Disease Epidemiology Collaboration equation (Levey et al. 2009).

*Definition of cut points for kidney effect markers.* Impaired kidney function was defined as having NAG, β_2_-microglobulin, or albumin above the 95th percentile for the study population in the nonpolluted Yantou area. We could not use the same cut points for 1998 and 2006 because the individuals were 8 years older in 2006 and renal markers are related to age. Therefore, we established different cut points for 1998 and 2006. Because the age distribution for the nonpolluted area differed from that of the two polluted areas (Table 1), we adjusted the distributions for all three biomarkers to the age distribution of the whole study group: Data were stratified into four age groups, and weights for each age group in the nonpolluted area were calculated by dividing the total proportion of individuals in an age group (all three areas) by the proportion of individuals in the nonpolluted area in that age group. In this way, a data set was created based on the nonpolluted area but with the same age distribution as the whole study group, which was used to establish the cut points for the kidney markers in 1998 and 2006. The weighted cut points for urinary NAG, β_2_-microglobulin, and albumin were 9.8 U/g creatinine, 0.890 mg/g creatinine, and 14.6 mg/g creatinine, respectively, in 1998; and 16.6 U/g creatinine, 1.028 mg/g creatinine, and 21.7 mg/g creatinine in 2006. Levels of these markers above the cut points were regarded as “elevated.” Values below the cut points were considered “normal.”

**Table 1:** Age distribution (%) by area among the residents followed up in 2006 (*n* = 412).

Age group (years) in 1998	Nonpolluted (*n* = 91)	Moderately polluted (*n* = 131)	Highly polluted (*n* = 190)
35–44		24.2		49.6		45.8
45–54		30.8		26.7		34.7
55–64		28.6		19.8		13.7
≥ 65		16.5		3.8		5.8

*Statistical methods.* Biochemical parameters that were not normally distributed were expressed as GMs with interquartile ranges (IQRs). We used the Wilcoxon signed-rank test to compare levels of biomarker values in 1998 with those in 2006, and the Mann-Whitney *U*-test to compare baseline characteristics of participants followed in 2006 and those lost to follow-up. Log-linear models were used to model the relation between prevalence of kidney damage (elevated NAG, β_2_-microglobulin, and albumin) and exposure (UCd in 1998). Pairwise comparisons were made between the prevalence of kidney damage in 1998 and 2006 in each exposure group. *p*-Values ≤ 0.05 were considered significant. All statistical analyses were performed using R, version 2.9.0 (http://www.r-project.org).

*Ethical considerations.* We complied with all applicable requirements of the international regulations during the study. Surveys in both 1998 and 2006 were carried out with the approval of the Ethics Committee of Fudan University and local authorities. All the participants gave written informed consent before the study.

## Results

At baseline in 1998, urinary albumin, β_2_-microglobulin, and NAG levels were all significantly higher in residents of the highly polluted area than in residents of the control area (Table 2; all *p* < 0.001). BCd decreased among all those surveyed in 2006, especially in the two polluted areas, whereas UCd decreased only in the highly polluted area (Table 2). Although both NAG and β_2_-microglobulin increased in individuals from all areas (*p* < 0.01), albumin tended to decrease, with a highly significant decline in the highly polluted area (*p* < 0.01).

**Table 2:** Exposure levels and renal indicators in 1998 and 2006 among residents in study areas (*n* = 412).

Nonpolluted area			Moderately polluted area			Highly polluted area		
Characteristic or indicator	1998	2006	1998	2006	1998	2006
*n* (% male)		91 (36.8)				131 (33.6)				190 (33.0)		
Median age (years) in 1998		53.0				46.0				46.5		
BCd (μg/L)		1.31 (0.75–2.19)		0.87 (0.57–1.46)*		3.78 (2.50–6.50)		1.80 (1.27–2.75)*		8.90 (4.97–13.6)		3.31 (2.25–5.15)*
UCd (μg/g creatinine)		1.79 (1.07–3.63)		2.31 (1.42–3.84)		3.62 (2.52–6.05)		3.79 (2.66–6.13)		11.6 (7.61–18.7)		8.97 (5.87–13.1)*
NAG (U/g creatinine)		1.80 (0.89–3.99)		7.92 (5.65–10.7)*		4.12 (2.02–11.3)		8.15 (5.77–10.7)*		7.64 (4.44–14.2)		11.8 (7.25–17.8)*
β_2_-Microglobulin (mg/g creatinine)		0.12 (0.07–0.24)		0.16 (0.09–0.26)		0.16 (0.10–0.31)		0.28 (0.17–0.44)*		0.28 (0.14–0.50)		0.42 (0.20–0.79)*
Albumin (mg/g creatinine)		3.08 (1.40–6.40)		2.84 (1.13–4.71)*		4.47 (2.10–9.30)		3.83 (1.33–9.44)		5.38 (2.48–11.9)		3.22 (1.28–7.22)*
Serum creatinine [μmol/L; median (range)]				65 (39–105)				66 (45–111)				70 (31–130)**
eGFR [mL/min/1.73 m^2^; median (range)]				92 (59–119)				93 (46–118)				92 (38–121)
Prevalence of hypertension (%)*a *				45.0				40.4				43.1
Values for BCd, UCd, NAG, β_2_‑microglobulin, and albumin are reported as GM (IQR). **a**Individuals with systolic pressure ≥ 140 mmHg or/and diastolic pressure ≥ 90 mmHg were classified as having hypertension. *p < 0.05 compared with 1998, by Wilcoxon signed-rank test. **p < 0.05 compared with residents of the nonpolluted area, by Mann-Whitney *U*‑test.												

In 2006, the serum creatinine concentration was higher in the highly polluted area than in the other two areas (*p* < 0.01), but there were no differences in estimated GFR (eGFR) between the nonpolluted area and polluted areas (Table 2). The eGFR in 2006 was significantly decreased only in those with the highest UCd in 1998 (> 20 μg/g creatinine) compared with participants who had < 2 μg/g creatinine UCd (Table 3).

**Table 3:** Serum creatinine and eGFR in 2006, by 1998 UCd group [median (range)].

1998 UCd group (μg/g creatinine)	*n*	Serum creatinine (μmol/L)	eGFR (mL/min/1.73 m^2^)
0–2		66		66 (45–100)		93 (60–119)
2–5		125		69 (47–112)		92 (52–118)
5–10		99		68 (31–113)		92 (55–120)
10–20		83		68 (39–112)		90 (46–121)
> 20		39		73 (45–130)		84 (38–109)*
*p < 0.05 compared with the 0–2 group, by Mann-Whitney *U*-test.						

The predictivity, on an individual basis, of the different biomarkers of kidney varied (Table 4). The probability of having an elevated β_2_-microglobulin (above the cut point) in 2006 was clearly associated with elevated β_2_-microglobulin in 1998 [odds ratio (OR) = 24.8; 95% confidence interval (CI): 11.2, 55.3). In contrast, much weaker significant associations were found between elevated albumin and NAG in 1998 and elevated values in 2006.

**Table 4:** The relationship between kidney function in 1998 and in 2006, as reflected by urinary NAG, β_2_‑microglobulin, and albumin.

Normal in 1998			Elevated in 1998		
Kidney function indicator	Normal in 2006 (*n*)	Elevated in 2006 (*n*)	Normal in 2006 (*n*)	Elevated in 2006 (*n*)	OR (95% CI)
NAG		247		37		92		36		2.6 (1.6, 4.4)
β_2_-Microglobulin		350		27		12		23		24.8 (11.2, 55.3)
Albumin		331		15		58		8		3.0 (1.2, 7.5)
Age-adjusted cut points were as follows: NAG, 9.8 U/g creatinine in 1998 and 16.6 U/g creatinine in 2006; β_2_-microglobulin, 0.890 mg/g creatinine in 1998 and 1.028 mg/g creatinine in 2006; albumin, 14.6 mg/g creatinine in 1998 and 21.7 mg/g creatinine in 2006.										

**Figure 1:**
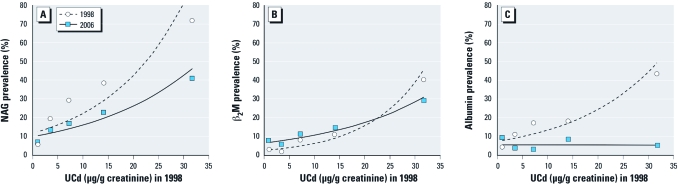
Prevalence of renal dysfunction in relation to 1998 UCd level shown by increased excretion of NAG (*A*), β_2_-microglobulin (β_2_M; *B*), and albumin (*C*) above the cut points.

Prevalence curves for kidney dysfunction in 1998 and 2006 in relation to UCd in 1998 are shown in Figure 1. The prevalence curve for elevated NAG in 2006 was slightly below the one for 1998, suggesting some recovery of kidney function after exposure was reduced, but the difference was not statistically significant (*p* = 0.06 for a decrease in the group with UCd > 20 μg/g creatinine). For β_2_-microglobulin the difference between the prevalence curves was far from statistically significant (*p* = 0.29 for a decrease in the group with UCd > 20 μg/g creatinine). In contrast, the prevalence curve for elevated albumin in 2006 was unrelated to UCd, despite a clear association in 1998 (*p* < 0.05 for a decrease in the three groups with UCd > 5 μg/g creatinine), suggesting that effects of exposure on albumin excretion were temporary.

**Figure 2:**
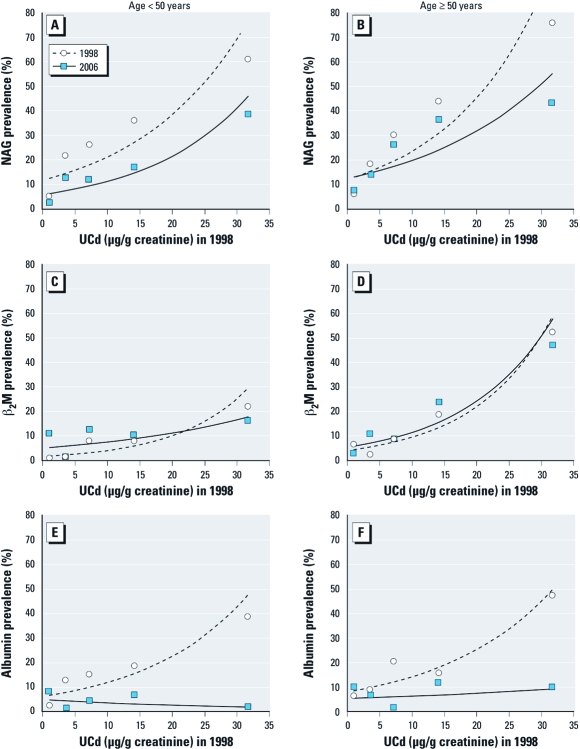
Prevalence of renal dysfunction in relation to 1998 UCd level in individuals < 50 years of age (*A*,*C*,*E*) and ≥ 50 years of age (*B*,*D*,*F*) shown by increased excretion of NAG (*A*, *B*), β_2_-microglobulin (β_2_M; *C*,*D*), and albumin (*E*,*F*) above the cut points.

**Figure 3:**
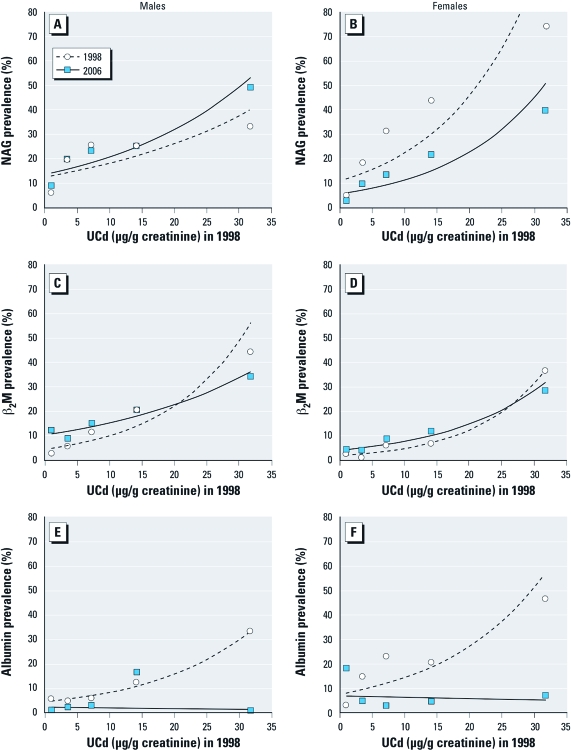
Prevalence of renal dysfunction in relation to 1998 UCd level in males (*A*,*C*,*E*) and females (*B*,*D*,*F*) shown by increased excretion of NAG (*A*,*B*), β_2_-microglobulin (β_2_M; *C*,*D*), and albumin (*E*,*F*) above the cut points.

The tendency toward recovery in β_2_-microglobulin in 2006 after exposure in 1998 appeared to be driven by recovery in individuals < 50 years of age at baseline, but sample sizes were small and differences were not statistically significant (Figure 2). Stratification by sex (Figure 3) did not indicate any major sex difference for β_2_-microglobulin or albumin. For NAG, however, the prevalence curve for men tended to be higher in 2006 than in 1998, whereas the opposite pattern appeared for women, suggesting the possibility of impairment in men but improvement in women. We also compared baseline characteristics between participants evaluated in 2006 and those lost to follow-up [see Supplemental Material, Table 1 (http://dx.doi.org/10.1289/ehp.1103699)]. Some differences appeared for age, β_2_-microglobulin, and albumin, but not in a systematic manner. Mean baseline UCd and BCd levels were comparable, although BCd in the moderately polluted area was statistically significantly lower in those lost to follow-up [GMs (IQRs) were 3.04 (1.88–5.00; see Supplemental Material, Table 1] compared with 3.78 (2.50–6.50) μg/L in the present study (Table 2). We also analyzed data for all subjects evaluated in 2006, including individuals who were > 80 years of age, had high blood pressure, or were on any physician-prescribed medication. The results were similar to those reported in Table 2 (see also Supplemental Material, Table 2).

## Discussion

In this study we observed that after cessation of exposure to contaminated rice, the Cd-related increase in urinary albumin was reversed. For the tubular markers after cessation of exposure, recovery was incomplete for NAG and there was little evidence of recovery of β_2_-microglobulin. In addition, the NAG status of an individual at baseline was a relatively weak predictor of elevated NAG at follow-up, whereas elevated β_2_-microglobulin at baseline was a strong predictor of renal tubular dysfunction 8 years later.

Repeated observations were made for > 400 individuals, and the 8-year time period appeared to be sufficient to detect changes in kidney function in association with the decrease in Cd exposure. To assure analytical quality, we incorporated reference materials for blood and urine into the analyses and made duplicate measurements of all biological indicators. In addition, several samples from the 1998 study were selected randomly and analyzed again together with the newly collected samples in 2006. The agreement between 1998 and 2006 results was very good [see Supplemental Material, Figure 1 (http://dx.doi.org/10.1289/ehp.1103699)].

The present study included > 50% of the original participants, and baseline characteristics of those who were lost to follow-up did not suggest that selection bias could explain the main findings of the study. A weakness of our study is that the prevalence analysis of renal dysfunction considered only whether individuals were above or below a cut point based on the distribution of biomarkers in the population with low exposure. In order to extract as much information as possible from our observations, it would be better to use continuous data for the kidney effect biomarkers. For example, Weaver et al. (2009) used generalized estimating equations to model change in renal effect markers and evaluated longitudinal associations between lead exposure and renal function in workers with occupational exposure. Although we reduced confounding by restricting the population according to age, blood pressure, and medication use, we did not perform multivariable models, and some residual confounding cannot be excluded.

In the present study, the decrease in BCd in both the moderately and highly polluted areas confirmed that there was a very substantial decrease in exposure in the two areas. BCd responded more to the reduction in exposure than did UCd. This is consistent with well-known differences in the kinetics of the two Cd exposure biomarkers, such that BCd mainly reflects recent exposure, whereas UCd is a better indication of long-term exposure and is thus used as a marker of body burden (Nordberg et al. 2007). In the residents of the nonpolluted area, the GM of UCd was about 10 times higher and that of BCd about 2–3 times higher than values reported for adults in Germany (Becker et al. 2002, 2003) and the United States (Centers for Disease Control and Prevention 2011), indicating a relatively high baseline Cd exposure in these areas in China. Based on urinary NAG and β_2_-microglobulin, kidney tubular function of the residents appeared to decline over time, despite reduction in Cd exposure (Table 2). This is consistent with the findings of Japanese studies with repeated sampling, which were interpreted as evidence of irreversible renal tubular injury in individuals environmentally exposed to Cd (Cai et al. 2001; Fan et al. 1998; Iwata et al. 1993; Kido et al. 1988).

Concerning blood pressure and the possible effects of mild Cd exposure, a number of previous studies reported variable results (Nordberg et al. 2007). Gallagher and Meliker (2010) reported a positive association between BCd and blood pressure among women. According to Lee et al. (2011), BCd may be related to an increased risk for hypertension among men in a general Korean adult population. In a study by Kacar Kocak et al. (2011), a rat experiment suggested that Cd exposure affected blood pressure by increasing plasma viscosity. In the present study, we excluded participants with hypertension to reduce potential confounding effects of high blood pressure, which is a risk factor for renal dysfunction.

The effect of age should be considered for kidney effect markers and has been taken into account in several epidemiological studies (Kobayashi et al. 2008; Moriguchi et al. 2003, 2005) and meta-analyses (Gamo et al. 2006; Omarova and Phillips 2007) of Cd. We used age-adjusted cut points to define abnormal kidney function to reduce the effect of age on our analysis of changes in kidney function over time.

In our stratified analyses for age ≥ 50 years versus < 50 years and for sex, we observed indications of a slight recovery in NAG and β_2_-microglobulin for some groups, whereas albumin showed a clear recovery (Figures 2 and 3).

Disease status is known to have great influence on GFR (Cirillo 2010). A community-based Japanese study (not related to Cd) revealed that individuals with baseline urinary β_2_-microglobulin > 300 μg/g creatinine had a greater decline in eGFR after 5 years (Kudo et al. 2011). Earlier studies of Cd-exposed workers indicated a relationship between BCd and decreased GFR. Jarup et al. (1995) found such a relationship among workers with BCd up to 14 μg/L, which is of the same magnitude as that found in the present study (interquartile range of 5–14 μg/L in the highly polluted area before reduction in exposure). In the present study, the renal filtration function of individuals in polluted areas in 2006 was at the levels of controls in that year, as indicated by eGFR (Table 2), but the individuals with the highest UCd in 1998 (> 20 μg/g creatinine) had a decreased eGFR in 2006 (Table 3). Unfortunately, we do not have eGFR data for 1998.

Mean levels of urinary albumin in 2006 decreased significantly from baseline levels among both men and women in the highly polluted area (*p* < 0.01; Table 2). Hotz et al. (1999) reported that urinary albumin decreased significantly in men, but not in women, after Cd body burden decreased slightly during a 5-year follow-up.

Previous studies on experimental animals (Bernard et al. 1992) and Cd-exposed workers (Bernard et al. 1988) have suggested that Cd-induced albuminuria might be due to loss of the glomerular polyanion charge and thus reduced electrostatic repulsion of negatively charged plasma proteins by the glomerular capillary wall. These studies and the study by Cardenas et al. (1991) have shown that loss of glomerular barrier function is reflected by a parallel loss of red blood cell charge and that these two phenomena are probably caused by a decrease of the sialic acid content of glomerular and red blood cell membranes. This is suggestive of a biochemical mechanism linked to circulating levels of Cd that might be reversible with a decrease in BCd, consistent with our findings. In contrast, effects of Cd on urinary excretion of β_2_-microglobulin and NAG are directly related to the accumulation of Cd in the proximal tubular cells, and there is no reason to believe that effects would be reversible after environmental exposure is reduced as long as the renal burden of Cd (as reflected by UCd) continues to be elevated.

The limited but statistically significant decrease in UCd in the highly exposed area (Table 2) may be partly related to the decrease in urinary albumin excretion, because Cd in blood plasma of animals has been shown to bind to albumin to a large extent (Nordberg et al. 1971). Obviously, when albumin excretion is changed, excretion of plasma Cd bound to this protein would be expected to change accordingly.

Hotz et al. (1999) reported that baseline levels of β_2_-microglobulin and NAG were not significant predictors of changes in urinary albumin 5 years after slightly reduced Cd exposure. Nishijo et al. (1994) identified urinary β_2_-microglobulin as an important prognostic factor, that is, an early index predictive of mortality. In our study we tested how strongly elevated levels of markers of renal tubular injury in 1998 predicted elevated levels of the same markers in 2006. The status of kidney function, as graded by dichotomous cut points, indicated for NAG that 2006 values were dependent on values in 1998 only to a limited degree (OR = 2.6). β_2_-Microglobulin, however, served as a good predictor for future results (OR = 24.8). The difference could be explained by the origins of urinary β_2_-microglobulin and NAG. β_2_-Microglobulin (11.8 kDa), normally found on the surface of nearly all nucleated cells and in small amounts in the blood and urine, is readily filtered through the glomerular membrane and almost completely resorbed by the renal proximal tubules (Wu et al. 2008). Impaired reabsorption leads to increased excretion of β_2_-microglobulin in urine. NAG, a lysosomal enzyme abundantly present in cells of renal proximal tubules, has a relatively high molecular weight of 130–140 kDa, which normally precludes its filtration by the glomerular basal membrane (Kawada et al. 1989; Price 1992). Because of NAG’s direct relevance to the integrity of proximal tubular cells, the release of NAG into the urine is an early sign of tubular membrane damage (Bernard et al. 1995; Jin et al. 1999). In addition, our recent animal study (Liang et al. 2010) confirmed increased hepatic and decreased urinary metallothioneins (a family of 6- to 7-kDa stress proteins incorporating a high content of cysteine and divalent metals) after cessation of oral Cd exposure, suggesting that urinary metallothionein could be another informative marker to evaluate recovery of Cd-mediated renal tubular dysfunction after a reduction in exposure.

A previous smaller study carried out over 3 years (1995–1998) in the same population described here indicated reversibility of β_2_-microglobulin and albumin excretion for individuals with UCd < 10 μg/g creatinine and progression at higher UCd (Wu et al. 2008). In the present study, we observed no such progression for β_2_-microglobulin at UCd > 10 μg/g creatinine; for albumin, a recovery was evident regardless of UCd value. Although we did not consider the relationship to BCd in the earlier study (Wu et al. 2008), it can be assumed that the 3-year period was too short to allow a sufficient decrease in BCd and liver Cd and an inflow of metallothionein-bound Cd from the liver to the kidney.

## Conclusions

Our findings suggest that a Cd-mediated increase in urinary albumin excretion (at the observed exposure levels) is a reversible effect that resolves after exposure is substantially reduced. For the markers of tubular effects, a tendency toward improvement, but not complete recovery, was observed for NAG, but only among women. Also, a small improvement for β_2_-microglobulin was suggested among those who were relatively young at baseline. In a previous 3-year study (Wu et al. 2008), we observed improvements for individuals with low exposure levels and increased impairment for those with high exposure levels. The lack of such increased impairment in the present 8-year study was probably related to the more pronounced decrease in BCd. Although increased NAG and β_2_-microglobulin at baseline both predicted increased levels in 2006, the association for β_2_-microglobulin was much stronger, suggesting that it may be more informative than NAG as a prognostic indicator for the individual’s future renal function.

## Supplemental Material

(160 KB) PDF
