# Supplementary material for: Renal Function after Reduction in Cadmium Exposure: An 8-Year Follow-up of Residents in Cadmium-Polluted Areas
Source: Environ Health Perspect. 2011 Oct 25;120(2):223–8. doi: 10.1289/ehp.1103699 (PMC3279438; doi:10.1289/ehp.1103699)
Supplement: (160 KB) PDF [file ehp.1103699.s001.pdf]

Supplemental Material

Renal Function after Reduction in Cadmium Exposure: An Eight-Year Follow-Up of Residents in Cadmium-Polluted Areas

Yihuai Liang, Lijian Lei, Johan Nilsson, Huiqi Li, Monica Nordberg, Alfred Bernard, Gunnar F. Nordberg, Ingvar A. Bergdahl and Taiyi Jin

Analytical performance. All the equipment was tested, and any contamination was below the limit of detection. For BCd and UCd, the limits of detection were 0.03 μg/L and 0.02 μg/L, respectively. No subjects had values below the detection limit. The method imprecision, calculated as the coefficient of variation for duplicate measurement was 8.3% for BCd and 8.0% for UCd. The analytical accuracy was checked against reference materials. For BCd, the Seronorm Trace Element Human Whole Blood (Nycomed Pharma AS, Oslo, Norway) was used, resulting in: 0.61 ± 0.15 μg/L (mean ± SD) to be compared with the recommended 0.7 μg/L. For UCd, the analyzed level of the Seronorm Trace Element Human Urine (Nycomed Pharma AS, Oslo, Norway) was 4.4 ± 0.25 μg/L *v.* recommended 5.0 μg/L. To check whether the determination of biological indicators in 2006 was in agreement with that in 1998, a number of 50 samples from the 1998 survey were randomly selected and measured again in the present study. The correlations between 1998 values and 2006 values for the same samples show that the results from these two determinations are comparable (See Figure 1 as below).

CKD-EPI (Chronic Kidney Disease Epidemiology Collaboration) equation :141*min(Scr/κ, 1)^α *max(Scr/κ, 1)^-1.209 * 0.993^Age × 1.018 [if female]*1.159[if black] (Scr is serum creatinine, κ is 0.7 for females and 0.9 for males; α is -0.329 for females and -0.411 for males; min indicates the minimum of Scr/κ or 1, and max indicates the maximum of Scr/κ or 1).

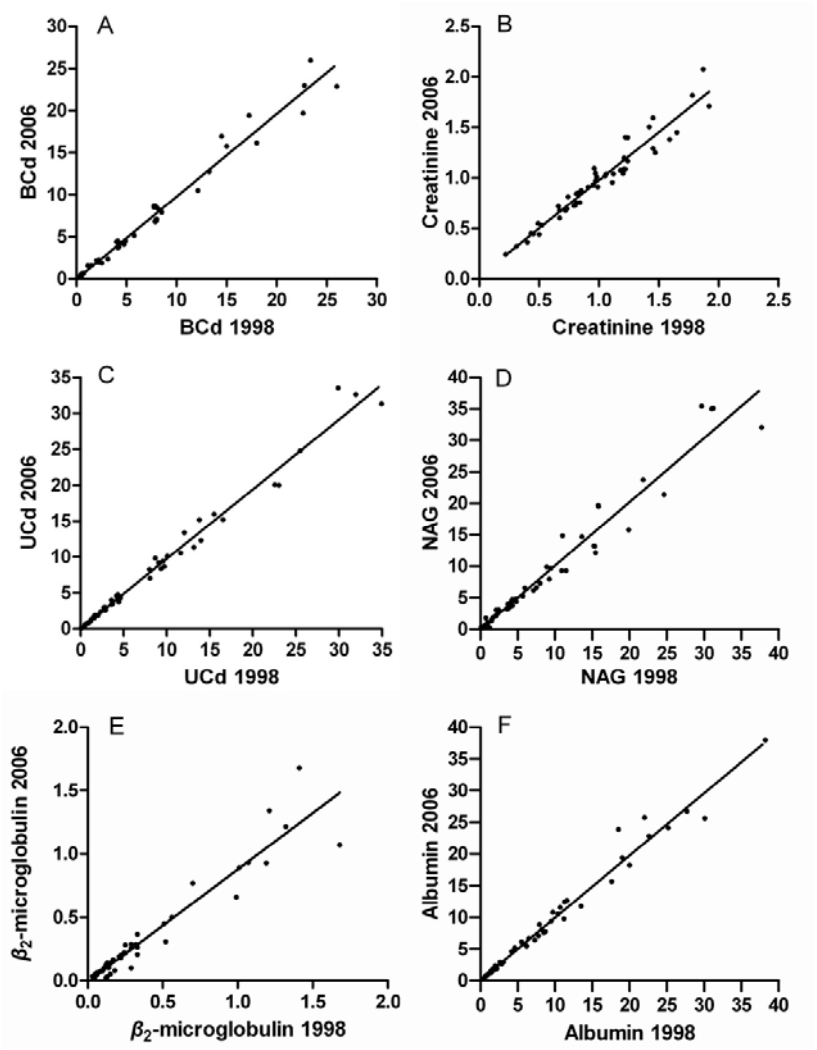

Supplemental Material, Figure 1. A number of 50 human blood and urine samples collected in the 1998 survey were selected randomly and measured again in 2006. Correlations of these repeated measurements between the 1998 values and their 2006 values were (A) blood cadmium (μg/L, $R^2 = 0.982$), (B) urinary creatinine (g/L, $R^2 = 0.937$), (C) urinary cadmium (μg/L, $R^2 = 0.984$), (D) urinary *N*-acetyl-β-D-glucosaminidase (U/L, $R^2 = 0.955$), (E) urinary β_2-microglobulin (mg/L, $R^2 = 0.891$) and (F) urinary albumin (mg/L, $R^2 = 0.971$).

Supplemental Material, Table 1. Levels of exposure and renal indicators in 1998 among residents lost to follow up in 2006 (N = 315).

Characteristic and indicator	Non-polluted area	p [a]	Moderately-polluted area	p [a]	Highly-polluted area	p [a]
N (Males %)	130 (43.1)		99 (33.3)		86 (52.3)	
Age (median, in 1998)	52.0	<0.05	47.0	0.993	55.5	<0.05
BCd (μg/L)	1.47 (0.95-2.19)	0.340	3.04 (1.88-5.00)	<0.01	9.20 (5.28-15.1)	0.678
UCd (μg/g crea)	1.75 (1.24-3.00)	0.262	3.45 (2.46-6.61)	0.996	10.7 (6.2-19.0)	0.531
NAG (U/g crea)	1.86 (1.04-5.04)	0.804	3.16 (1.53-10.6)	0.337	9.70 (4.31-24.4)	0.123
β_2-microglobulin (mg/g crea)	0.19 (0.09-0.34)	0.058	0.17 (0.10-0.29)	0.456	0.49 (0.14-1.39)	<0.01
Albumin (mg/g crea)	3.03 (1.38-5.75)	0.708	4.02 (2.00-8.60)	0.337	7.82 (3.85-18.2)	0.029

BCd, blood cadmium; UCd, urinary cadmium; NAG, *N*-acetyl-β-D-glucosaminidase; creatinine, crea. These data were reported as geometric mean and interquartile range.[a] Mann-Whitney U test: comparison of the corresponding values in 1998 between those followed in 2006 and those lost in 2006.

Supplemental Material, Table 2. Levels of exposure and renal indicators in 1998 and 2006 among all residents followed up in 2006 (N = 475).

	Non-polluted area		Moderately-polluted area		Highly-polluted area	
Characteristic and indicator	1998	2006	1998	2006	1998	2006
N (Males %)	123 (34.1)		144 (33.3)		208 (37.5)	
Age (median, in 1998)	58.0		46.0		47.0	
BCd (μg/L)	1.35 (0.75-2.13)	0.91 (0.59-1.46) [a]	4.15 (2.53-6.63)	1.82 (1.25-2.74) [a]	9.00 (5.00-14.1)	3.36 (2.30-5.15) [a]
UCd (μg/g crea)	1.91 (1.19-3.64)	2.40 (1.45-4.10)	3.62 (2.54-6.00)	3.81 (2.66-6.14)	11.4 (7.31-17.9)	8.98 (5.85-13.4) [a]
NAG (U/g crea)	1.97 (0.95-4.48)	9.33 (6.01-12.5) [a]	3.84 (1.88-11.2)	8.24 (6.01-10.8) [a]	7.47 (4.29-14.1)	11.9 (7.32-17.8) [a]
β_2-microglobulin (mg/g crea)	0.14 (0.07-0.26)	0.20 (0.10-0.34) [a]	0.16 (0.10-0.28)	0.28 (0.17-0.42) [a]	0.28 (0.14-0.52)	0.44 (0.21-0.80) [a]
Albumin (mg/g crea)	3.09 (1.50-6.40)	2.93 (1.31-6.48)	4.56 (2.03-10.15)	3.98 (1.40-9.63)	5.31 (2.50-11.8)	3.57 (1.43-8.64) [a]
Serum creatinine (μmol/L)		66 (60-114)		66 (45-111)		71 (31-130) [b]
eGFR (mL/min/1.73 m^2)		89 (38-119)		93 (46-121)		91 (38-121)

BCd, blood cadmium; UCd, urinary cadmium; NAG, *N*-acetyl-β-D-glucosaminidase; creatinine, crea; eGFR, estimated glomerular filtration rate. BCd, UCd, NAG, β_2-microglobulin and albumin were reported as geometric mean and interquartile range, and serum creatinine and eGFR as median and range.[a] Wilcoxon Signed Ranks Test: comparison of values between 1998 and 2006 among those followed in 2006, $p < 0.05$.[b] Mann-Whitney U test: compared with "non-polluted", $p < 0.05$.
